# Mandibular Fracture in A 12‐Hour‐Old Neonate: Successful Conservative Management With Excellent Long‐Term Functional and Craniofacial Growth Outcomes

**DOI:** 10.1002/ccr3.73415

**Published:** 2026-08-26

**Authors:** Symon Guthua, Wambeti Twahir, Krishan Sarna, Khushboo Sonigra, Jane Kabutu

**Affiliations:** ^1^ Unit of Oral and Maxillofacial Surgery, Oral Pathology and Oral Medicine, Department of Dental Sciences, Faculty of Health Sciences University of Nairobi Nairobi Kenya; ^2^ Department of Human Anatomy and Medical Physiology, Faculty of Health Sciences University of Nairobi Nairobi Kenya; ^3^ Department of Obstetrics and Gynecology, Faculty of Health Sciences University of Nairobi Nairobi Kenya; ^4^ The Kenya Society of Anesthesia (KSA), Faculty of Health Sciences University of Nairobi Nairobi Kenya

**Keywords:** long‐term follow‐up, mandibular growth, neonatal mandibular fracture, neonatal oral and maxillofacial trauma, perinatal trauma

## Abstract

Neonatal mandibular fractures are exceptionally rare but can be successfully managed with timely conservative treatment and anatomical reduction. Despite severe birth‐related maxillofacial trauma, the neonatal mandible demonstrates remarkable regenerative capacity, allowing excellent long‐term mandibular growth, occlusion, function, and facial aesthetics into adulthood.

## 1 Introduction

Neonatal birth trauma is an important cause of morbidity worldwide, with a disproportionately higher incidence reported in low‐ and middle‐income countries, particularly in Africa [1]. Several maternal, fetal, and delivery‐related factors have been associated with an increased risk of birth trauma, including precipitous labor, fetal malpresentation, and instrumental delivery [1, 2]. Despite these risks, neonatal head and neck trauma is uncommon, accounting for only approximately 0.82% of all birth injuries [3]. Among these injuries, nerve damage is the most frequently encountered, followed by vascular injuries, whereas fractures constitute only a minority of cases and rarely involve the head and neck region [4].

Mandibular fractures in the neonatal period are exceptionally rare, with only nine cases reported in the English‐language literature since 1949 [5]. Most reported cases have been attributed to traumatic delivery or low‐impact trauma in the perinatal period [5, 6]. The rarity of these injuries is largely explained by the unique anatomical characteristics of the neonatal mandible, including its elasticity, incomplete ossification, developing tooth buds, and ongoing craniofacial growth, all of which confer substantial resistance to fracture [7]. Although these features provide protection against injury, they may also complicate diagnosis and treatment when fractures do occur.

Reported neonatal mandibular fractures predominantly involve the symphyseal region and commonly present with oral bleeding, hematoma formation, feeding difficulties, segmental mobility, or displacement of the mandibular fragments [8]. Although the neonatal mandible possesses considerable regenerative potential, management remains challenging because treatment must achieve adequate fracture stabilization while minimizing the risk of adverse effects on future mandibular growth, dental development, and occlusal function. Owing to the extreme rarity of these injuries, current management strategies are largely derived from isolated case reports and small case series, and evidence‐based treatment recommendations remain limited [6]. Consequently, each additional case contributes valuable information to the existing body of literature.

We present an exceptionally rare case of a mandibular fracture in a 12‐h‐old neonate associated with a severe, full‐thickness lower lip laceration following a birth‐related fall. Despite the severity of the injury, conservative management resulted in excellent long‐term functional, aesthetic, and developmental outcomes. The extended follow‐up into adulthood of this case provides one of the first opportunities to assess mandibular growth and craniofacial development following neonatal mandibular trauma.

## 2 Case Presentation

### 2.1 Case History and Examination

A female neonate weighing 3180 g was delivered at full‐term gestation through an unassisted spontaneous vaginal delivery at home following precipitous labor. The mother was a 16‐year‐old, Para 1 + 0, with an uneventful antenatal course and no significant past medical history. According to the reported history, the infant sustained facial trauma during delivery, following an accidental fall during the second stage of labor. There was profuse oral bleeding and progressive swelling of the lower face. Initial stabilization was performed at a peripheral health facility, after which the patient was referred to Kenyatta National Hospital (KNH) for definitive assessment and management.

At presentation at KNH, 12 h after birth, the neonate continued to experience bleeding and an inability to feed effectively. There was no history of loss of consciousness, seizures, apnoeic episodes, cyanosis, or respiratory compromise. On admission, the neonate was alert and hemodynamically stable with a heart rate of 148 beats/min, respiratory rate of 42 breaths/min, temperature of 36.8°C, and oxygen saturation of 98% on room air with no signs of respiratory distress. A comprehensive secondary survey revealed no evidence of concomitant injuries involving the scalp, skull, cervical spine, thorax, abdomen, pelvis, or extremities.

### 2.2 Differential Diagnosis, Investigations, and Treatment

**FIGURE 1:**
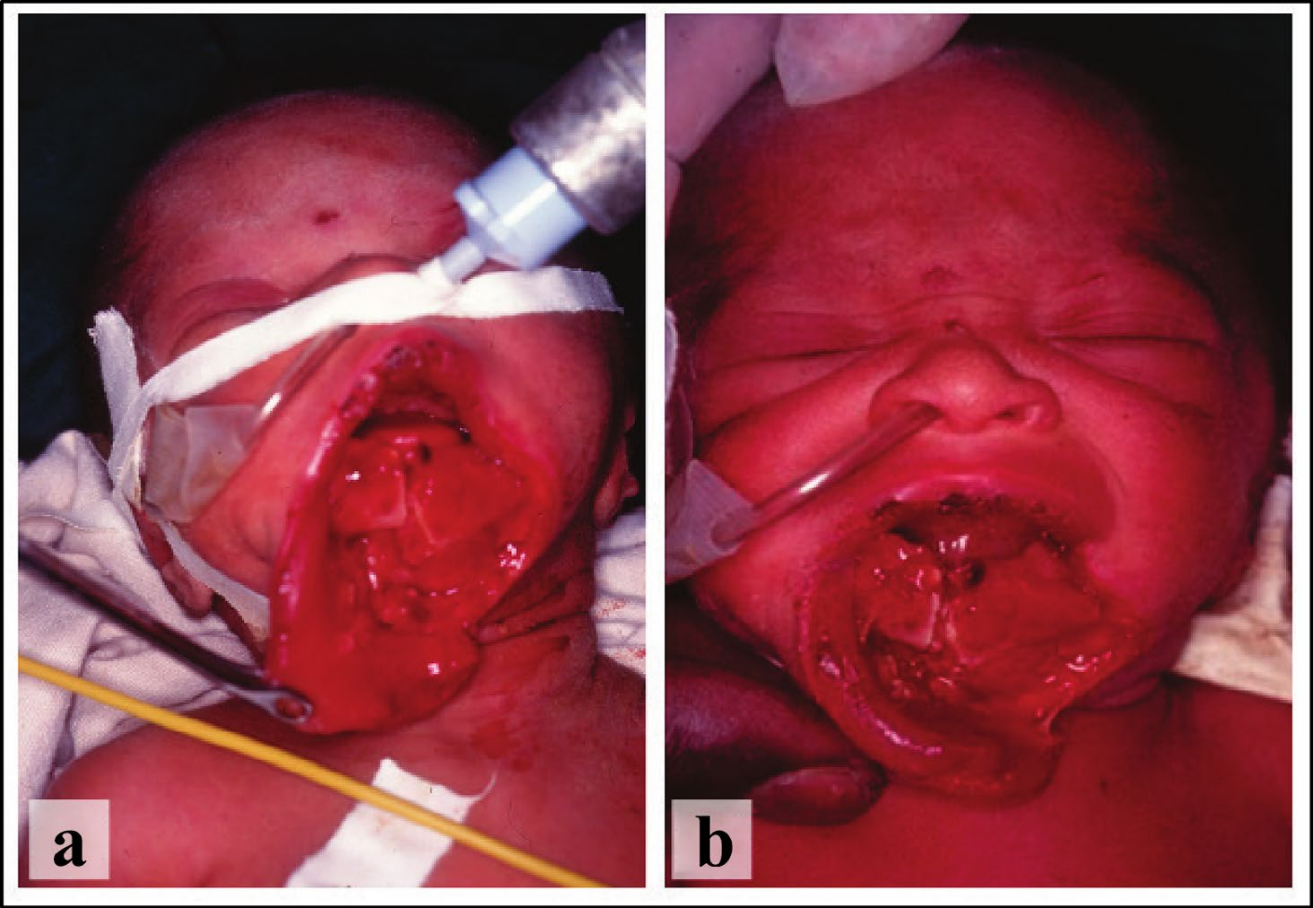
Appearance of the neonate at presentation. Note the symphyseal mandibular fracture accompanied by a severe laceration of the lower lip.

Extraoral examination revealed moderate swelling of the lower third of the face with mild facial asymmetry. A full‐thickness laceration of the lower lip and anterior perioral soft tissues was present on the left side, resulting in an extensive degloving‐type defect with near‐complete avulsion of the lower lip and exposure of the underlying mandibular symphysis with diastasis (Figure 1). The tongue was displaced posterosuperiorly by the unstable mandibular fragments. There was no evidence of acute airway compromise. Neurological examination was otherwise unremarkable, with preservation of age‐appropriate neonatal reflexes.

Following a multidisciplinary assessment involving the oral and maxillofacial surgery, pediatric, and anesthesia teams, a conservative management plan was developed. This decision was based on the patient's age, fracture pattern, absence of significant airway compromise, and the considerable osteogenic and remodeling potential of the neonatal mandible.

**FIGURE 2:**
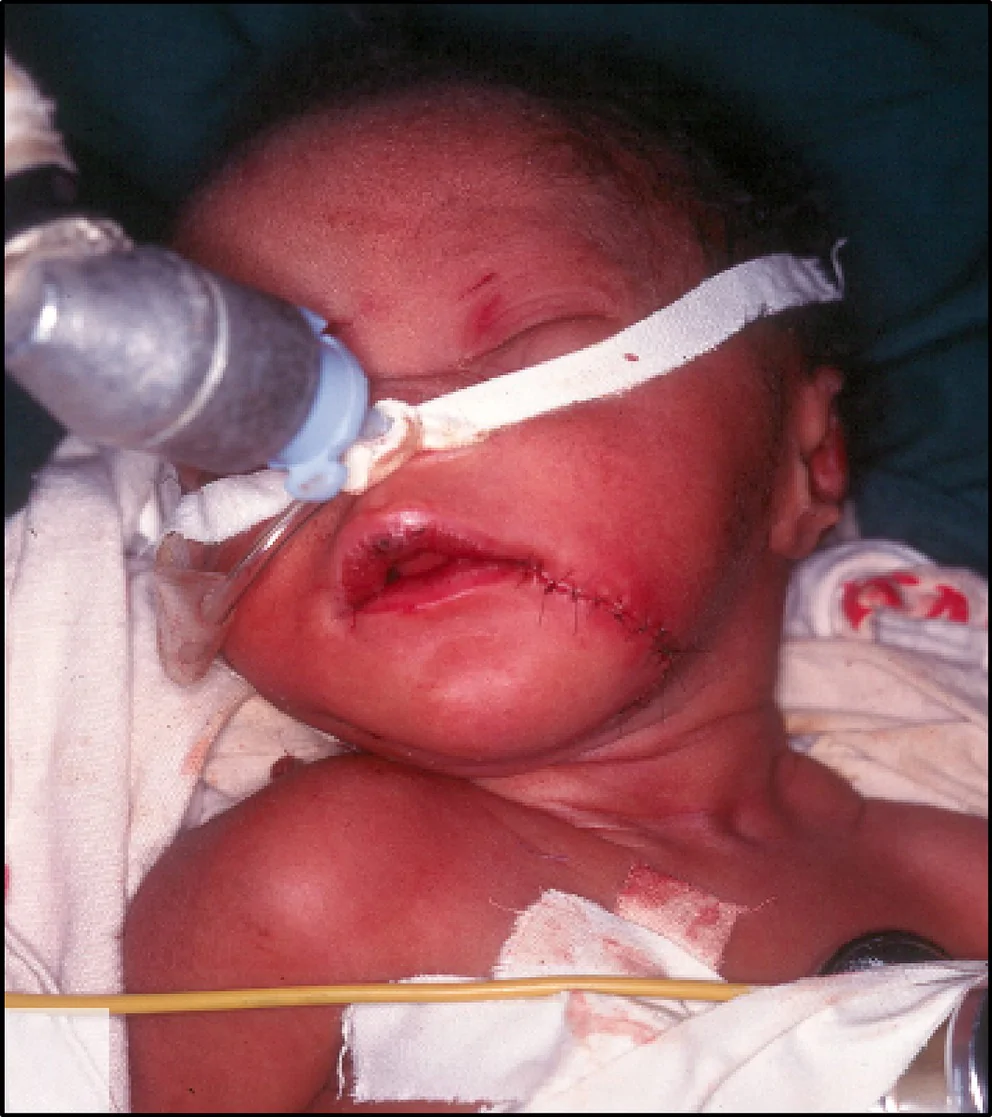
Immediate postoperative appearance following fracture reduction and primary closure of the lower lip and perioral soft‐tissue defects.

Under general anesthesia, the fracture site was accessed through the existing soft‐tissue defects, which provided adequate visualization of the displaced mandibular segments without the need for additional surgical incisions. Manual manipulation was used to achieve anatomical reduction of the displaced fracture fragments. Stabilization was then achieved using four interrupted 3–0 polyglactin (Vicryl) sutures, with two placed on the lingual aspect and two on the buccal aspect of the fracture. This technique was feasible because of the relatively soft and malleable nature of the neonatal mandible. The reduced segments were further stabilized by restoring the surrounding periosteal and soft‐tissue envelope with 3–0 and 4–0 polyglactin (Vicryl) sutures, respectively, thereby re‐establishing continuity of the functional soft‐tissue matrix around the fracture site. Closure of the soft‐tissue injuries was completed in layers, using 5–0 polyglactin (Vicryl) sutures for the oral mucosa, and interrupted 6–0 non‐absorbable nylon sutures for the skin and lower lip (Figure 2). Particular care was taken during reconstruction to achieve accurate alignment of the orbicularis oris muscle and the lower lip sphincter to restore functional continuity. The patient was monitored postoperatively in the neonatal intensive care unit (NICU).

### 2.3 Outcome and Follow‐Up

The postoperative course was uneventful. The patient remained hemodynamically stable with no evidence of airway compromise, wound infection, or fixation failure. Progressive reduction in facial swelling and improvement in mandibular stability were observed during the early postoperative period. Soft‐tissue healing and clinical stability proceeded satisfactorily, and the patient was discharged following confirmation of adequate feeding on expressed breast milk. Direct breastfeeding was avoided for the first 8 weeks postoperatively to minimize stress on the healing mandible. Breastfeeding was thereafter introduced without complication.

**FIGURE 3:**
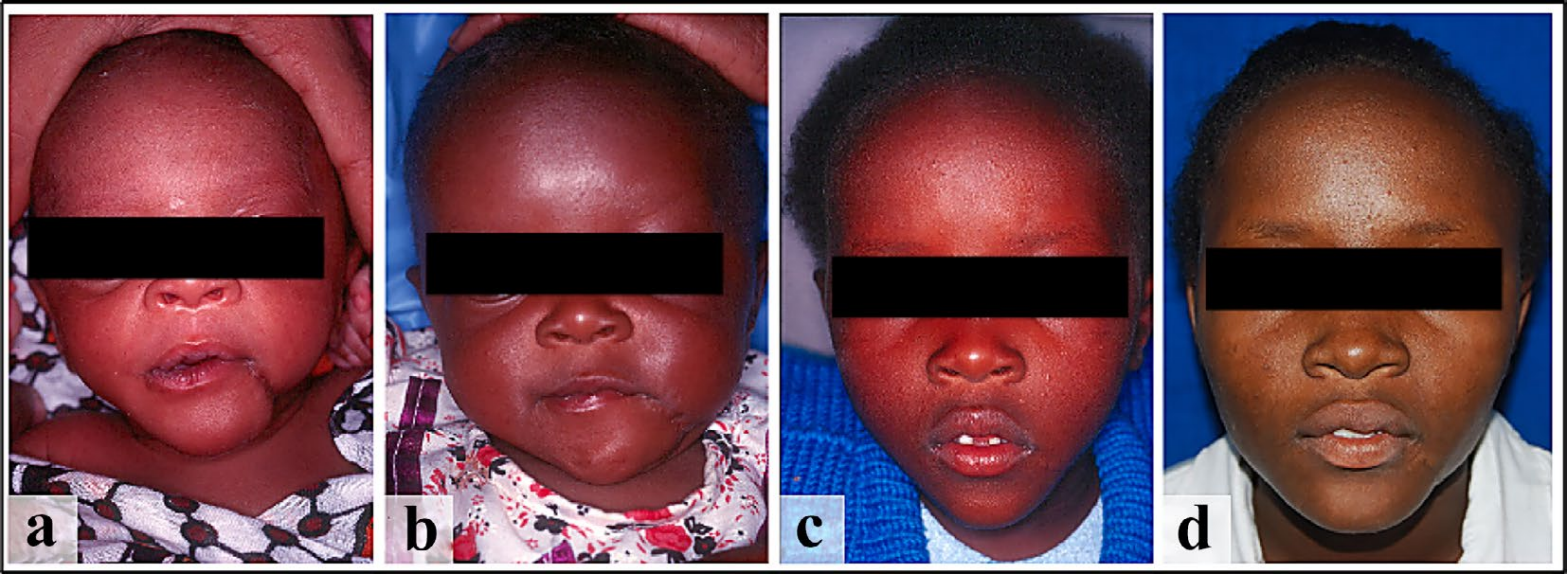
Serial clinical photographs demonstrating progressive facial growth and healing following management of a neonatal symphyseal mandibular fracture and extensive lower lip injury. From left to right: Follow‐up at 3 months, 1 year, 4 years, and 18 years of age.

**FIGURE 4:**
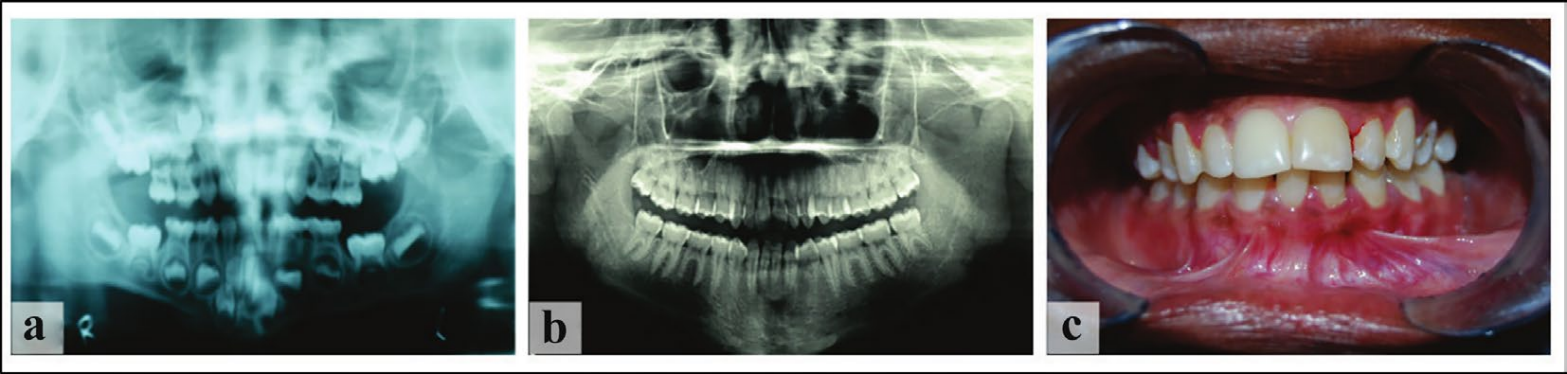
Long‐term follow‐up demonstrating favorable mandibular growth and dental development. Left: Panoramic radiograph at 4 years of age. Center: Panoramic radiograph at 18 years of age. Right: Intraoral appearance at 18 years showing stable occlusion and satisfactory healing of the lower anterior vestibular soft tissues.

At the 3‐month follow‐up visit, the surgical wounds demonstrated satisfactory healing. Clinical examination revealed improved mandibular stability and resolution of the tongue displacement. There were no postoperative complications after removal of the skin nylon sutures. The patient was followed up in early childhood, then late adolescence, and into adulthood. She demonstrated normal mandibular growth, facial development, dental eruption, and oral function, with no evidence of facial asymmetry, growth disturbance, or temporomandibular joint dysfunction. (Figure 3). The scar was pronounced in the early days but faded over time, becoming barely noticeable in adulthood, with a small linear scar extending from the left angle of the mouth to the chin. Intraorally, she had a scar in the left labial sulcus in the canine region (Figure 4). At 18 years of age, clinical evaluation revealed satisfactory facial symmetry, stable occlusion, normal mandibular function, and an acceptable cosmetic outcome, with no residual functional deficits. Overall, long‐term follow‐up demonstrated satisfactory functional, aesthetic, and developmental outcomes (Figures 3 and 4). A recent review of the patient at age 29 showed a healthy, well‐adjusted young woman who reported no long‐term adverse effects and was satisfied with her functional and aesthetic outcomes.

## 3 Discussion

The present case is notable for several reasons. First, the injury occurred in a neonate only 12 h after birth, making it the youngest reported patient with a mandibular fracture. Second, the fracture was accompanied by extensive lower lip injury, resulting in direct exposure of the fractured mandibular segments, significant hemorrhage, and feeding impairment. Finally, and most importantly, follow‐up extending to 29 years of age provided a rare opportunity to evaluate the long‐term effects of neonatal mandibular trauma and its management on craniofacial growth and development.

The overarching goal in the management of neonatal mandibular fractures is to restore function and facial symmetry while minimizing disruption to growth centers and developing tooth buds [9]. The literature consistently emphasizes that treatment should be as conservative and noninvasive as possible to avoid long‐term complications, such as growth disturbances or dental anomalies [10]. In some cases, observation alone may suffice for non‐displaced or greenstick fractures, given the high osteogenic potential and rapid healing observed in neonates [10, 11]. When intervention is required for displaced or unstable fractures, closed reduction techniques are preferred. These include acrylic splints secured with circummandibular wiring or bridle wiring using orthodontic ligature wire, methods that provide stabilization without extensive surgical exposure or risk to tooth buds [10, 11]. Open reduction and internal fixation are rarely indicated in neonates but may be necessary only for severely displaced or comminuted fractures [12].

In the present case, the extensive soft‐tissue injury provided direct access to the fracture site, allowing anatomical reduction without additional surgical exposure. Fracture stabilization was achieved using sutures placed through the surrounding soft tissues and periosteal envelope, thereby avoiding hardware placement and minimizing disruption of developing anatomical structures. To our knowledge, the use of resorbable sutures as the primary method of fracture stabilization in a neonatal mandibular fracture has been scarcely reported, with only one other report on the same [9]. A major strength of this approach was its ability to achieve a satisfactory reduction while adhering to the biological principles of preserving growth potential and minimizing iatrogenic injury. However, this technique may be applicable only in carefully selected cases and should not be considered a universal alternative to other fixation methods.

Despite the severity of the initial injury, fracture healing was uneventful, and no disturbances in mandibular growth, facial symmetry, dental development, occlusion, or temporomandibular joint function were identified during follow‐up. These findings are particularly significant because severe mandibular injuries in early life can lead to growth disturbances, facial asymmetry, temporomandibular joint dysfunction, or secondary craniofacial deformity if inadequately managed [10]. Although children generally experience lower rates of infection, non‐union, and malunion than adults because of their remarkable healing capacity, the long‐term effects of neonatal mandibular trauma remain poorly understood [12].

A major limitation of the existing literature is the scarcity of long‐term outcome data, with most reported cases documenting only short‐term follow‐up [10]. Consequently, the true impact of neonatal mandibular fractures on subsequent craniofacial growth and development remains uncertain. The present case provides rare longitudinal evidence demonstrating that satisfactory functional, aesthetic, and developmental outcomes can be achieved following early management of a displaced neonatal mandibular fracture. These findings suggest that when anatomical reduction is achieved and biological tissues are preserved, normal craniofacial development can be maintained despite severe neonatal mandibular trauma. The principal lesson from this case is that conservative treatment strategies can achieve excellent long‐term outcomes, emphasizing the importance of balancing fracture stabilization with preservation of the developing craniofacial skeleton.

This case demonstrates that favorable long‐term craniofacial growth, dental development, and functional outcomes can be achieved following early conservative management of a displaced neonatal mandibular fracture, even in the presence of severe associated soft‐tissue injury. The 29‐year follow‐up provides rare evidence supporting the effectiveness of growth‐preserving treatment strategies in this uniquely vulnerable patient population. More importantly, it highlights the need to balance immediate fracture stabilization with preservation of future craniofacial development, recognizing that treatment decisions made in the neonatal period may have lifelong consequences.

## Author Contributions

**Symon Guthua:** conceptualization, data curation, visualization, writing – original draft, methodology, investigation, writing – review and editing, resources, project administration. **Khushboo Sonigra:** data curation, visualization, writing – original draft, methodology, project administration, writing – review and editing, resources. **Wambeti Twahir:** conceptualization, data curation, writing – review and editing, project administration, resources, methodology, investigation, visualization. **Krishan Sarna:** writing – original draft, visualization, writing – review and editing, project administration, methodology, resources, data curation. **Jane Kabutu:** writing – review and editing, resources, methodology, visualization, data curation, investigation, validation.

## Funding

The authors have nothing to report.

## Consent

Written informed consent for publication of this manuscript and the accompanying clinical images was obtained from the patient.

## Conflicts of Interest

The authors declare no conflicts of interest.

## Data Availability

Data available from the authors upon reasonable request.
